# Mid-phase pinpoint hyperfluorescent spots on fundus fluorescein angiography in acute central retinal artery occlusion – a novel imaging finding

**DOI:** 10.1186/s40942-023-00478-5

**Published:** 2023-07-05

**Authors:** Rubble Mangla, Ramesh Venkatesh, Vishma Prabhu, Isha Acharya, Ashit Handa, Atul Thadani, Aishwarya Joshi, Naresh Kumar Yadav, Jay Chhablani

**Affiliations:** 1 Dept. of Retina and Vitreous, Narayana Nethralaya, #121/C, 1st R Block, Chord Road, Rajaji Nagar, 560010 Bengaluru, Karnataka India; 2 Medical Retina and Vitreoretinal Surgery, University of Pittsburgh School of Medicine, 203 Lothrop Street, Suite 800, Pittsburg, PA 15213 USA

**Keywords:** Artery occlusions, Rouleaux formation, RBC aggregation, Fundus fluorescein angiography, Pinpoint hyperfluorescent spots

## Abstract

**Purpose:**

To describe the development and outcome of mid-phase pinpoint hyperfluorescent spots (MPHS) on fundus fluorescein angiography (FFA) in acute (< 7-day symptom onset) central retinal artery occlusion (CRAO) patients.

**Methods:**

This retrospective study included acute CRAO patients who underwent multimodal imaging utilizing optical coherence tomography (OCT) and FFA between June 2017 and January 2023. The correlation of FFA images with the OCT images in various stages and severity of CRAO were studied.

**Results:**

Twenty-three FFA studies on 23 patients with acute CRAO were included. In 11 (48%) cases, an important finding noted on FFA was the presence of single or multiple MPHS with adjacent minimal late vascular leakage. Of these 11 cases, eight (73%) were males and median age was 41 years (inter quartile range: 33–55 years). Visual acuity ranged from ‘light perception’ to 6/18, and these patients presented anytime on the same day to seven days after symptom onset. On OCT, three eyes had severe CRAO, seven eyes had moderate CRAO, and one eye had mild CRAO. MPHS were primarily observed at the posterior pole and more frequently observed in moderate CRAO severity. During follow-up, the MPHS and retinal vessel staining on FFA disappeared as the CRAO showed signs of resolution.

**Conclusion:**

MPHS at the posterior pole on FFA in acute CRAO patients could indicate a more severe occlusion and poor visual outcomes, even after treatment. This finding is most likely caused by red blood cell aggregation or rouleaux formation.

**Trial registration number:**

Not applicable.

**Supplementary Information:**

The online version contains supplementary material available at 10.1186/s40942-023-00478-5.

## Introduction

A clinical diagnosis of CRAO is based on typical patient symptoms such as sudden onset painless vision loss, fundus findings such as extensive retinal whitening and cherry red spot, retinal vessel attenuation, and preservation of optic nerve head perfusion, and OCT characteristics such as inner retinal layer opacification and thickening [1]. In routine clinical practice, fundus fluorescein angiography (FFA) is still underutilized by many retina specialists for acute CRAO. Gong et al. examined the relationship between central visual impairment and the characteristics of FFA in patients with CRAO and identified three angiographic patterns, namely poor perfusion, exudative, and mixed, based on the arm-retina time and retinal vessel and optic disc leakage [2]. The damage to vision caused by the exudative type of CRAO was less severe than that caused by the poor perfusion and mixed types.

In our limited experience with FFA in acute CRAO cases, we observed in the middle phase of the angiogram, pinpoint hyperfluorescent spots primarily at the posterior pole, along with subtle late vascular leakage. There is limited literature on these hyperfluorescent pinpoint spots. To learn more about hyperfluorescent spots on FFA and its association with the severity of CRAO and visual prognosis, we aimed to examine the FFA findings in patients with acute CRAO and a symptom onset duration of less than 7 days. In addition, this study seeks to determine the etiopathogenesis of this imaging finding observed on FFA in acute CRAO.

## Methods

In this study, we retrospectively reviewed the medical records of patients who were clinically diagnosed with acute CRAO with a symptom onset duration of less than 7 days. Acute occlusion of the central retinal artery was defined as a recent history of sudden, painless vision loss accompanied by retinal thickening and whitening, retinal vessel attenuation, and a cherry-red spot in the macula. Patients who visited the retina clinic between June 2017 and January 2023 were included in the study. In addition, only patients who had undergone an invasive fundus fluorescein angiography and OCT imaging were included in the subsequent analysis. In a case of acute CRAO, a FFA was performed at the discretion of the clinician in order to document the arm-retina time and thus estimate the location and severity of occlusion and final visual outcome. In addition, it was performed to rule out partial or total ophthalmic artery occlusion. The clinician considered repeat FFA primarily when the OCT findings worsened or to examine the changes in the previous FFA findings. The local Institutional Review Board/Ethics Committee approved the research because it adhered to the Declaration of Helsinki’s principles. Prior to the procedures, separate informed patient consent was obtained for invasive retinal imaging with FFA.

Demographics, associated systemic history, corrected distance visual acuity and time interval between onset of symptoms and presentation to clinic were all recorded during the presentation and follow-up visits. Clinical features were documented using the Optos Optomap Daytona Panoramic 200Tx (Daytona, Optos®, UK) or Topcon TRC-50Dx (Topcon Medical Systems Inc, Oakland, NJ, USA) machines, and OCT was performed using the spectral domain Spectralis (Heidelberg Engineering, Heidelberg, Germany). The cSLO-based Spectralis machine was also used for FFA imaging. All patients had a complete hemogram, random blood sugar for diabetes, blood pressure check-up, lipid profile, serum homocysteine levels, carotid doppler ultrasound, cardiac evaluation, and stroke work-up.

Macular volumetric assessments were performed using 512 A-scans per line with a 30° scanning area and 25-line horizontal raster scans centered on the fovea. In addition, each eye obtained a 12-line foveal-centered radial scan. The presence of inner retinal layer hyperreflectivity, inner retinal layer thickening and loss of individual inner retinal layer stratification, presence of cystoid macular edema, and neurosensory detachment were all observed on OCT. The severity of acute CRAO was classified based on OCT findings into mild, moderate, and severe occlusion [3]. The early angiography images were evaluated for the presence of the cilioretinal artery and for documenting the arm-retina time and in the middle phase to identify the pinpoint hyperfluorescent spots at the posterior pole. Further comparisons were conducted between the two groups of eyes: (A) eyes that displayed pinpoint hyperfluorescent spots in the middle phase of the fluorescein angiogram; and (B) eyes that did not display such spots.

### Statistical tests

All data were analysed using GraphPad Prism version 9.5.0 (730) for Windows, GraphPad Software, San Diego, California USA, www.graphpad.com. Only statistical tests related to the analysis of non-parametric data were used in this study. Quantitative data between the 2 groups of cases were analysed using the Mann-Whitney U test. Chi-square test was used to compare the categorical data between 2 groups. P values < 0.05 were considered statistically significant.

## Results

This retrospective study identified 48 eyes in 48 cases of acute CRAO with a symptom onset duration of less than seven days. In 23 (48%) of the 48 cases with acute CRAO, fluorescein angiography information was available. Eleven (48%) of the 23 eyes with acute CRAO demonstrated the presence of single or multiple, pinpoint hyperfluorescent spots in the mid-phase of the angiogram. We shall henceforth refer to them as mid-phase pinpoint hyperfluorescent spots or MPHS. Table 1 compares the patient and ocular characteristics between CRAO eyes showing the MPHS and those not showing them. There were no significant differences in the patient and ocular characteristics between the two groups of cases in terms of age, gender, presence of cilioretinal artery, severity of CRAO and arm-retina time.

**Table 1:** Comparisons in the patient and ocular characteristics in acute CRAO eyes between those not demonstrating and those demonstrating the mid-phase pinpoint hyperfluorescent spots on FFA at presentation

	CRAO eyes without MPHS (n = 12)	CRAO eyes with MPHS (n = 11)	P value
Age (Median, IQR) [years]	52.5 (48–70.5)	41 (33–55)	0.062
Gender (Males) (n, %)	9 (75)	8 (73)	> 0.999
Visual acuity range	LP negative – 6/60	LP positive – 6/18	
Duration of symptoms (days)	5.583 ± 1.775	2.182 ± 1.093	0.075
Presence of cilioretinal artery (n, %)	6 (50)	5 (45)	> 0.999
Mild CRAO (n, %)	3 (25)	1 (9)	0.59
Moderate CRAO (n, %)	4 (33)	7 (64)	0.22
Severe CRAO (n, %)	5 (42)	3 (27)	0.67
ART (seconds)	29.96 ± 11.06	21.76 ± 9.25	0.062

Abbreviations: CRAO – central retinal artery occlusion; FFA – fundus fluorescein angiography; MPHS – mid-phase pinpoint hyperfluorescent spots; LP – light perception; IQR – interquartile range; ART – arm-retina time

Further, we analysed in detail 15 FFA image sets from 11 cases of acute CRAO with MPHS. Only these 11 acute CRAO patients with MPHS were included in subsequent demographic and ocular data reporting. Eight (73%) males and three (27%) females participated in the study. In 8 (73%) cases, the right eye was affected, and in 3 (27%) cases, the left eye was affected. The median age of the patients was 41 years, and the interquartile range was 33 to 55 years. 82% (n = 9) of patients with CRAO had at least one associated systemic condition, such as diabetes mellitus, valvular heart disease, hypertension, internal carotid artery occlusion, stroke, or hyperhomocysteinemia. Visual acuity ranged from ‘light perception’ to 6/18, and patients presented to the clinic anytime from the same day to seven days after the onset of symptoms. No patient had a significant refractive error, and the anterior segment of all 11 patients was unremarkable. In these cases, FFA was performed on the same day or within two days of presentation to the retina clinic. All the 11 eyes with MPHS showed adjacent minimal late vascular leakage on FFA. Based on OCT classification, three eyes had severe CRAO, seven eyes had moderate CRAO, and one eye had mild CRAO. One patient (case 4) presented with cystoid macular edema and neurosensory detachment, while another patient (case 3) exhibited only a small subfoveal pocket of subretinal fluid. Table 2 provides a summary of the demographic and clinical characteristics of all eleven cases on the day of presentation.

**Table 2:** Patient characteristics, ocular findings, and retinal imaging features in patients with acute CRAO and MPHS on FFA:

Case No.	Age	Sex	Eye	Presenting Visual acuity	Symptom onset (days)	Associated systemic disease	CRAO severity	CME (Y/N)	NSD (Y/N)	FFA done after (days)	Arm-retina time (seconds)	Cilioretinal artery (Y/N)	Final visual acuity	Total follow-up period (months)
1	53	M	LE	CFCF	5	Hyperhomocysteinemia, hyperlipidaemia	Mild	N	N	0	32.3	N	CFCF	2
2	55	M	RE	HM	0	Hypertension	Severe	N	N	1	26.8	N	CF ½ MT	1
3	33	M	RE	3/60	7	Vasculitis	Moderate	N	Y	2	10.9	Y	1/60	2
4	37	F	RE	LP	0	Heart disease	Moderate	Y	Y	0	14.9	Y	6/60	6
5	41	M	RE	HM	0	Hypertension, hypercholesterolemia	Moderate	N	N	1	26.3	N	CF 1 MT	2
6	56	M	RE	6/75	2	Hypertension, ICA occlusion	Severe	N	N	0	14	N	6/75	1
7	20	M	LE	HM	0	Vasculitis	Moderate	N	N	1	27	N	3/60	4
8	34	M	RE	CF ½ MT	3	Nil	Severe	N	N	2	16.5	Y	CF ½ MT	1
9	76	F	LE	CF ½ MT	0	Hypertension, ICA occlusion	Moderate	N	N	1	39.7	Y	CF ½ MT	1
10	21	F	RE	CF ½ MT	1	Nil	Moderate	N	N	0	12.5	N	CF ½ MT	1
11	46	M	RE	6/18	3	Diabetes Mellitus	Moderate	N	N	0	18.5	Y	6/6	5

Abbreviations: CRAO – central retinal artery occlusion; MPHS – mid-phase pinpoint hyperfluorescent spots; FFA – fundus fluorescein angiography; M – male; F – female; RE – right eye; LE – left eye; CFCF – counting fingers close to face; HM – hand motions; LP – light perception; CF – counting fingers; ICA – internal carotid artery; CME – cystoid macular edema; NSD – neurosensory detachment; Y – yes; N - no

**Fig. 1:**
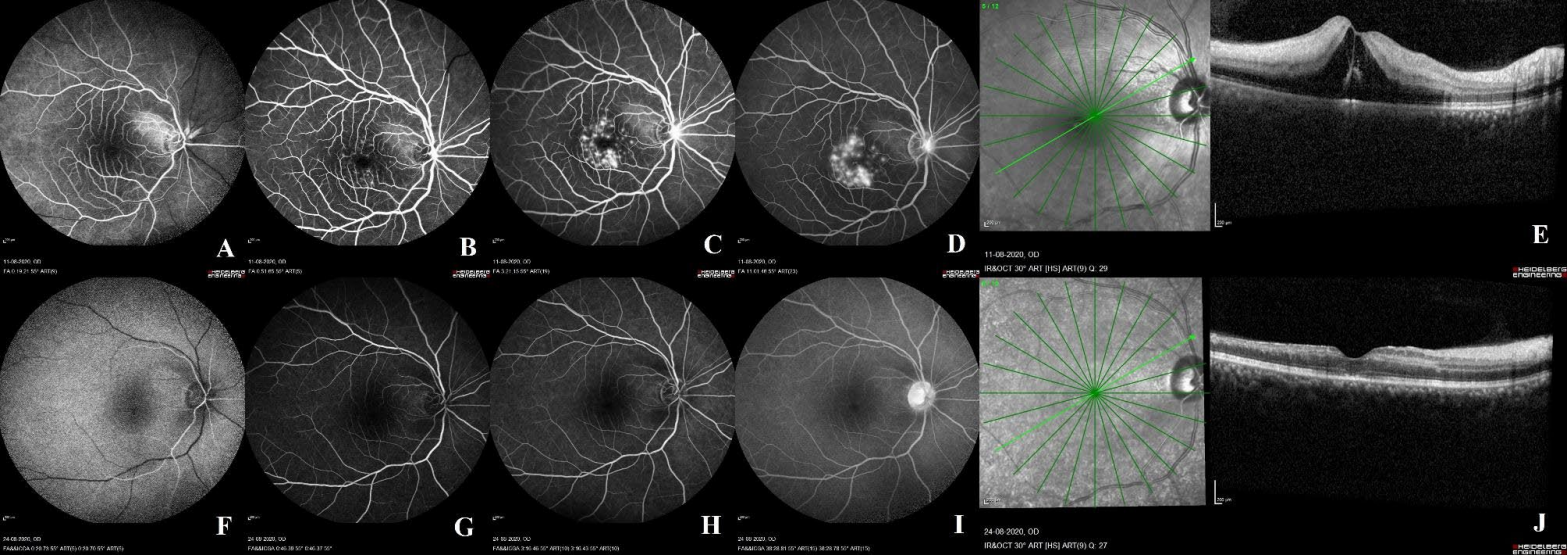
Fundus fluorescein angiography (FFA) and optical coherence tomography (OCT) features of case 4: A 37-year-old female with a history of valvular heart disease presented to the retina clinic with sudden vision loss in the right eye over the course of the last few hours. Her right eye presented with light perception vision. On the day of presentation, an OCT scan of the right eye revealed moderate severity of CRAO, cystoid macular edema, and a small subfoveal pocket of neurosensory detachment. On the same day, FFA was performed, revealing the presence of a patent cilioretinal artery and an arm-retina time of 14.9 s. Multiple hyperfluorescent pinpoint spots were identified in the FFA’s middle phase. They were associated with minimal vascular leakage and staining in the final phase of the FFA (**A-E**). Thirteen days later, OCT and FFA imaging of the retina was repeated. The OCT scan revealed thinning of the inner retinal layers, resolution of the cystoid macular edema, and neurosensory detachment. During this imaging session, the hyperfluorescent pinpoint spots observed in the previous FFA images had disappeared (**F-J**).

**Fig. 2:**
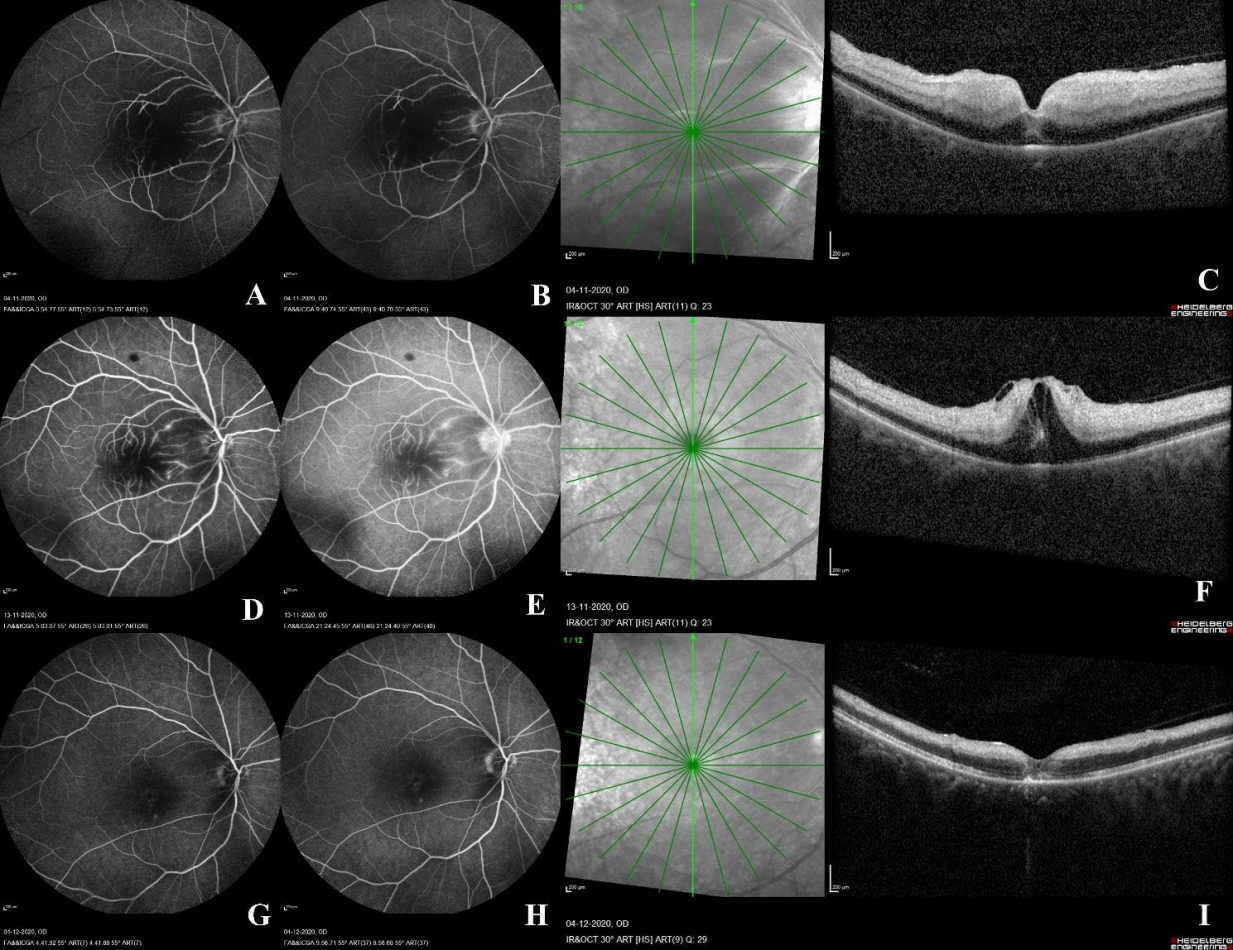
Fundus fluorescein angiography (FFA) and optical coherence tomography (OCT) features of case 5: A 41-year-old male with a systemic history of hypertension and hypercholesterolemia presented to the retina clinic with sudden vision loss in the right eye over the course of the last few hours. His right eye vision was hand motions. On the day of presentation, an OCT scan of the right eye revealed moderate severity of CRAO with inner retinal hyperreflectivity and thickening. On the following day, FFA was performed, revealing the absence of a patent temporal cilioretinal artery and an arm-retina time of 26.3 s. Multiple hyperfluorescent pinpoint spots were identified in the FFA’s middle phase. They were associated with minimal vascular leakage and staining in the final phase of the FFA (**A-C**). Nine days later, OCT and FFA imaging of the retina was repeated. The OCT scan revealed inner retinal hyperreflectivity, thickening and loss of retinal layer stratification suggestive of a severe grade of occlusion in the central retinal artery. This was associated with the development of cystoid macular edema, neurosensory detachment and internal limiting membrane separation as well. Similar to the previous FFA visit, the FFA at this visit showed similar findings of middle phase pinpoint hyperfluorescent spots with late staining and vascular leakage (**D-F**). Thirty days from the initial presentation, retinal imaging with OCT and FFA were repeated. OCT showed thinning of the inner retinal layers, resolution of the cystoid macular edema, neurosensory detachment and internal limiting membrane separation. During this imaging session, the hyperfluorescent pinpoint spots observed in the previous FFA images had disappeared (**G-I**).

FFA was repeated in three cases (cases 4, 5, and 10) during subsequent follow-up visits. The purpose of repeating the angiographies was to compare the FFA image changes with the findings from the previous visit and to correlate them with the OCT image changes as well. Within two weeks, the second round of angiographies were performed on all three patients. In two of the three cases, the MPHS seen on the initial angiographies had disappeared (case 4 and 10) (Fig. 1). In case 5, the MPHS were still visible, and the OCT performed on the same day revealed a progression from moderate to severe CRAO severity. Thirty days after the initial presentation, FFA was repeated for the third time on this patient. This time, the MPHS were absent (Fig. 2). At the time of MPHS resolution, OCT scans of all three cases revealed an atrophy of the inner retinal layers. Last visit visual acuity ranged from “counting fingers close to face” to 6/6, and follow-up duration ranged from one month to six months.

## Discussion

This study describes the presence of a peculiar FFA finding of MPHS in the eyes of patients with acute CRAO. To the best of our knowledge, no previous literature reports have documented or described this finding. The typical characteristics of these MPHS are their location predominantly at the posterior pole, their prevalence in moderate to severe grades of acute CRAO, and their emergence during the middle phase of the fluorescein angiogram with late staining and subtle adjacent perivascular leakage. These MPHS persist until the severity of occlusion exist and disappear in the resolving stages of CRAO. There are no significant relationships between the presence of MPHS and age of presentation, symptom duration, CRAO severity or arm-retina time delay.

In the case of an ophthalmic emergency, such as acute CRAO, the primary goal of treatment is to quickly relieve the obstruction and restore retinal perfusion. As a result of its invasive nature and limited ability to influence the treatment decision, FFA is a relatively uncommon imaging technique in clinical practice. Consequently, retinal imaging findings with FFA in acute CRAO are rarely described in the scientific literature. This could be the primary reason why MPHS on FFA in acute CRAO cases have yet to be described in the literature. Gong et al. identified three types of CRAO patterns on FFA, including poor perfusion, exudation, and mixed patterns, based on the arm-retina time and retinal vascular leakage [2]. These findings have demonstrated significant advantages for predicting the visual prognosis of CRAO patients.

The occurrence of multiple hyperfluorescent spots at the posterior pole beginning in the middle phase of the angiogram and extending until the late phase of the acute stages was an interesting and significant finding in the current series. The exact nature of these MPHS is unknown. One may consider them to be true emboli (fibrin-platelet or cholesterol) released from the thrombus responsible for the obstruction of the retinal artery. This may be unlikely, however, in the absence of non-perfusion distal to these hyperfluorescent spots. Red blood cell (RBC) aggregates in the retinal circulation is a possible alternative explanation for these hyperfluorescent spots. In the aftermath of an acute occlusion of the central retinal artery, blood flow distal to the occlusion becomes stagnant or diminished. Blood flow is affected more often as occlusion grades increase. The surface properties of RBCs and fibrinogen concentrations could also lead to RBC clustering [4]. This leads to RBC aggregation and rouleaux formation [5]. As indicated by FFA, the formation of rouleaux damages the endothelium of the capillaries, resulting in breakdown of the blood retinal barrier and leakage from the capillaries into the intraretinal space. Rouleaux formation is more prevalent in the retinal capillary circulation as a result of the smaller vessel diameter [6]. This also suggests that the spots are more likely to be observed at the retinal level than at the choroidal level, since only the retinal arteriolar and capillary anatomy is suitable for such a staining. This may not be possible in the choroidal circulation due to the larger caliber and high flow characteristics of the choroidal vessels [7]. In this series, we observed MPHS with a higher severity grade of CRAO and at the posterior pole, where retinal capillary density is greatest and capillary vessel caliber is smallest. In the milder forms of acute CRAO, there does not appear to be any blood flow obstruction that would lead to RBC aggregation and rouleaux formation. In the most severe stages of acute CRAO, extensive retinal whitening and thickening may obscure the visibility of the MPHS beneath the retina on FFA. Thus, MPHS were observed more frequently on FFA in the moderate severity of occlusion than in the milder and more severe grades of disease.

The aggregation of RBCs is a reversible phenomenon. The MPHS in our current series of cases disappeared in subsequent FFA studies as the CRAO showed signs of resolution. This supports our contention that the MPHS seen on FFA are most likely RBC aggregates similar to the ‘box-carring’ of larger retinal arteries seen on clinical examination and FFA. The MPHS could reveal the severity of the occlusion and the visual prognosis. The presence of the MPHS, however, had no effect on the treatment decision.

Single or multiple hyperfluorescent spots at the posterior pole are seen in a number of choroidal diseases, including central serous chorioretinopathy, Vogt-Koyanagi Harada syndrome, sympathetic ophthalmia, posterior scleritis, and occasionally due to drusen staining in age-related macular degeneration [8]. Aside from the abnormal retinal pigment epithelium detected on OCT, the time of appearance of the hyperfluorescent spot could be crucial in making a diagnosis. The hyperfluorescence spots seen in acute CRAO appear in the middle phases of the angiogram and remain hyperfluorescent due to RBC aggregate staining of the dye. In contrast, hyperfluorescence seen in choroidal pathologies begins early in the angiogram and continues to increase in size and intensity until late in the angiogram, indicating leakage.

This study has several limitations. In this study, only a small proportion of cases initially underwent FFA. Second, only patients who had undergone invasive FFA were included in the analysis, which introduces the possibility of selection bias. As the FFA was recommended at the discretion of the clinician and the study design was retrospective, this could not have been avoided. Third, the timing of the FFA was inconsistent in all instances, which could be attributed to its retrospective nature, which is one of its major limitations. Fourth, the FFA result was not correlated with the treatment modality or treatment outcomes; therefore, it does not contribute to the treatment decision. On the other hand, research on this topic is limited. This study describes a novel imaging finding on FFA in acute CRAO cases and offers a possible explanation for its presence. The discovery could be an indirect predictor of visual prognosis in cases of acute CRAO.

To summarize, MPHS at the posterior pole on FFA in acute CRAO cases could imply greater occlusion severity and, as a result, poor visual outcomes even after treatment. The most likely pathogenesis for this finding is RBC aggregation. Future research would be needed to determine whether FFA is truly a necessary investigation for acute CRAO.

## Electronic supplementary material

Below is the link to the electronic supplementary material.

Supplementary Material 1

## Data Availability

The datasets used and/or analysed during the current study are available from the corresponding author on reasonable request.

## Abbreviations

CRAO: Central retinal artery occlusion
FFA: Fundus fluorescein angiography
MPHS: Mid-phase pinpoint hyperfluorescent spots
OCT: Optical coherence tomography
RBC: Red blood cell

## Declarations

### Animal research

This article does not contain any studies with animals performed by any of the authors.
