# Supplementary material for: Mid-phase pinpoint hyperfluorescent spots on fundus fluorescein angiography in acute central retinal artery occlusion – a novel imaging finding
Source: Int J Retina Vitreous. 2023 Jul 5;9:40. doi: 10.1186/s40942-023-00478-5 (PMC10321000; doi:10.1186/s40942-023-00478-5)
Supplement: Supplementary file 1 — Supplementary Material 1 [file 40942_2023_478_MOESM1_ESM.docx]

Reviewer reports:

EIC: this article provides interesting data. However, in order to be acceptable for publication, some improvements are required.

Reply: Thank you very much for the favourable comments and suggestions.

Reviewer #1: Dear authors,

Congratulations on this interesting case series presenting a novel finding on fundus fluorescent angiography.

Reply: We thank the reviewer for providing important and valuable suggestions to this manuscript.

Please consider the following comments and suggestions.

The introduction is clearly written and explains the importance of the study.

Reply: Thank you.

The methods section is overall clear. However, it would benefit from some minor adjustments:

1.    Page 5, line 1 mentions two groups of cases that were compared. Later in the results section, we realize that they refer to CRAO eyes with the MPHS finding and those not showing them. I would suggest defining the groups also in the methods section.

Reply: The groups are now defined in the methodology section of the manuscript.

The results section presents relevant findings. However, one issue may be pointed out:

2.    Page 5, line 43 says, "patients presented to the clinic anytime from the same day to ten days after symptom onset". However, in the introduction and methods sections, the authors stated that the study aimed to examine the records of patients with a symptom onset of less than seven days.

Reply: Thank you for pointing this out. It was a typographical error and is now rectified in the revised manuscript.

In the discussion section, the authors provide well-structured arguments about the possible etiology of the findings. Nonetheless, some conclusions seem precipitated and need to be addressed:

3.    Page 7, line 55 states, "the MPHS indicate the severity of the occlusion and the visual prognosis". The analyzed data cannot support this statement - MPHS were seen in eyes with mild, moderate, and severe CRAO. The severity distribution between the two groups (MPHS present vs. absent) was not statistically significant.

4.    Page 8, line 34 stated that "MPHS (…) imply greater occlusion severity and, as a result, poor visual outcomes even after treatment". Similarly, to item 3, it is not possible to affirm that the observed finding correlates to the severity of the disease. Also, the study does not include data on visual outcomes after treatment. These statements could be, instead, presented in terms like "could imply" or "could be" to indicate that they are a supposition based on the mechanisms of etiopathogenesis, and not a study finding.

Reply: For points 3 and 4, we accepted the suggestions made by the reviewer and changed them in the revised manuscript.

5.    Page 8, line 19 acknowledges the study's limitations. The study has an important potential bias arising from the fact that only patients who had undergone an invasive fundus fluorescein angiography were included in the analysis. This could lead to a selection bias since FFA was performed at the clinician's discretion (and not randomly within the sample of patients with CRAO), which could imply significant differences between cases that were conducted with further imaging and those that were not, compromising the generalizability of the findings. Evidently, as a retrospective study, one cannot avoid this; however, I believe it should be acknowledged in the discussion.

Reply: This point has been acknowledged in the limitation’s section of the manuscript.

Reviewer #2: Many thanks for having me look at this interesting manuscript.

Reply: Thank you for your valuable comments.

However, i would like to suggest some changes

-page 3 row 55-59: I don't think it's necessary to explain the CRAO

Reply: Introduction has been modified and the suggestions given by the reviewer has been accepted.

-statistical analysis: Have you considered any logistic regression analysis (for example, with OCT features), or do you prefer to simply have a descriptive study?

Reply: We prefer to keep the study as a descriptive one.

-page 5 row 8-16 i prefer this part in the method sections

Reply: Suggestions made by the reviewer has been accepted.

-page 6 row 34-40: I prefer in present

Reply: Suggestions made by the reviewer has been accepted.

-page 7 row 5: replace not known with unknown

Reply: Suggestions made by the reviewer has been accepted.

Improve the physio pathological explanation of RBC aggregate (is there some literatures?)

Reply: There have been a few modifications in the revised manuscript. They are marked in red in the revised manuscript.
